# Potential Anti-*Mycobacterium tuberculosis* Activity of Plant Secondary Metabolites: Insight with Molecular Docking Interactions

**DOI:** 10.3390/antiox10121990

**Published:** 2021-12-14

**Authors:** Manu Kumar, Sandeep Kumar Singh, Prem Pratap Singh, Vipin Kumar Singh, Avinash Chandra Rai, Akhileshwar Kumar Srivastava, Livleen Shukla, Mahipal Singh Kesawat, Atul Kumar Jaiswal, Sang-Min Chung, Ajay Kumar

**Affiliations:** 1 Department of Life Science, College of Life Science and Biotechnology, Dongguk University, Seoul 10326, Korea; manukumar007@gmail.com; 2 Division of Microbiology, Indian Agricultural Research Institute, Pusa, New Delhi 110012, India; sandeepksingh015@gmail.com (S.K.S.); lshukla65@gmail.com (L.S.); 3 Centre of Advance Study in Botany, Banaras Hindu University, Varanasi 221005, India; prempratapsingh31@gmail.com (P.P.S.); vipinks85@gmail.com (V.K.S.); 4 Institute of Plant Sciences, Agriculture Research Organization, Volcani Center, Rishon LeZion 7505101, Israel; avinashchandraraigene29@gmail.com; 5 Plant Cell Biotechnology Department, CSIR-Central Food Technological Research Institute, Mysore 570020, India; akhileshwar.kumar2@gmail.com; 6 Faculty of Agriculture, Sri Sri University, Cuttack 754006, India; mahipal.s@srisriuniversity.edu.in; 7 School of Computational and Integrative Sciences, Jawaharlal Nehru University, New Delhi 110067, India; atulj91@gmail.com; 8 Department of Postharvest Science, Agriculture Research Organization, Volcani Center, Rishon LeZion 7505101, Israel

**Keywords:** plant secondary metabolites, antioxidant activity, drug discovery, multi-drug resistance (M.D.R.), molecular docking, tuberculosis

## Abstract

Tuberculosis (TB) is a recurrent and progressive disease, with high mortality rates worldwide. The drug-resistance phenomenon of *Mycobacterium tuberculosis* is a major obstruction of allelopathy treatment. An adverse side effect of allelopathic treatment is that it causes serious health complications. The search for suitable alternatives of conventional regimens is needed, i.e., by considering medicinal plant secondary metabolites to explore anti-TB drugs, targeting the action site of *M. tuberculosis*. Nowadays, plant-derived secondary metabolites are widely known for their beneficial uses, i.e., as antioxidants, antimicrobial agents, and in the treatment of a wide range of chronic human diseases (e.g., tuberculosis), and are known to “thwart” disease virulence. In this regard, in silico studies can reveal the inhibitory potential of plant-derived secondary metabolites against *Mycobacterium* at the very early stage of infection. Computational approaches based on different algorithms could play a significant role in screening plant metabolites against disease virulence of tuberculosis for drug designing.

## 1. Introduction

Plants produce a diverse range of secondary metabolites (SMs), due to various physiological and metabolic processes, which, since ancient times, have served as “raw” material for enhancing the immune system and in the treatment of various human ailments. Currently, more than 75% of the global population, especially developing countries, rely on plant metabolites or natural products for their primary health treatments [1]. In addition, more than 25% of modern drugs and 60% of total anti-cancer drugs are derived from plant secondary metabolites, directly or indirectly [2,3]. The diverse range of functional groups of secondary metabolites offer opportunities to search or target the molecular sites of pathogens, which is an essential condition for drug discovery [4].

Tuberculosis (TB), one of the most contagious global diseases, is considered one of the top ten most lethal diseases worldwide [5]. TB, being a fatal infectious disease, has shown its devastating nature by infecting over 10.0 million (~9.0–11.1 million) people worldwide in 2018. However, the burden of TB is increasing continuously, with approximately 5 to >500 new cases each year (per millions) of the global population; the global average is around 130 cases. Noteworthy, a higher severity of TB has been reported in developing countries. It is worth noting that the high impact of TB on the host immune system may increase the incidence of the disease [6]. There was a dramatic decline in the number of TB cases in the 1950–1970s, likely due to the discovery of an effective antimycobacterial agent viz. ethambutol, isoniazid, pyrazinamide, and rifampicin. However, presently, there is a massive upsurge in TB cases throughout the world due to the emergence of multi-drug resistance (MDR), extensively drug resistance (XDR), and total drug resistance (TDR) in mycobacteria [7]. The misuse of antimycobacterial drugs has led to the prevalence of these “outdriven” TB conditions (MDR and XDR). As per an estimate, in the global context, there was a substantial increment of up to 186,772 MDR-TB cases observed in 2018 compared to the previously reported 160,684 cases in 2017. The resistance developed in *Mycobacterium tuberculosis* (Mtb) has caused inefficiency in the first-line and (some of the) second-line drugs commonly used for treatment. Additionally, popular second-line drugs, such as ethionamide, capreomycin, and kanamycin, employed to cure MDR/XDR-TB, have some safety concerns, with only a 50% cure rate. While safer second-line drugs (e.g., ofloxacin and norfloxacin) have better efficiency, the drawback is they are expensive. Therefore, there is an urgent need to develop new, affordable, and effective anti-tuberculosis drugs with unique drug targets, multi-domain inhibitory effects, possessing fewer and negligible side effects [8].

Plants, being significant reservoirs of various biologically active compounds, play an essential role in curing several human diseases. Plant-derived phytochemicals have a long history of providing much-needed novel therapeutics [9,10]. The major portion of the globe, i.e., Africa, Asia, Latin America, and the Middle East, with 70–95% of the entire population, use traditional medicine for their primary healthcare needs [11]. In addition, several plant secondary metabolites, i.e., alkaloids, coumarins, flavonoids, polyphenols, terpenoids, triterpenoids, quinines, plumbagin, maritinone, 3,3’-biplumbagin, aloe-emodin, epigallocatechin, and umckalin, have been widely exploited for their broad-spectrum activities against various human diseases.

## 2. Plant Secondary Metabolites as Antioxidant and Antimycobacterial Agents

Secondary metabolites (SMs) produced by plants are defined as a different group of natural intermediary metabolic products that are not obligatorily required for the vegetative growth of plants [12]. These small molecules are derived mainly from the primary metabolites, in which some are nitrogen-containing alkaloids (e.g., amino acids, amines, cyanogenic glycosides, and glucosinolates), non-nitrogen compounds polyphenols, terpenoids, flavonoids, steroids, lignin, and tannins [13]. Since ancient times, plant extracts have been used as (an easy source of) antibiotics/antioxidants and applied as crude/extract against bacterial or fungal infections, with minimal side effects [14]. Out of all the plant-synthesized metabolites, alkaloids and polyphenols have potent antimicrobial and antioxidant properties. Alkaloids have a possible role in the development of antibiotics, whereas plenty of polyphenols provide a wide range of antioxidant properties that eventually establish the basis of antimicrobial activity [15]. Extreme environmental changes and various physiological or metabolic processes of the body can generate free radicals, which are continuously neutralized by antioxidant molecules. The optimum requirements of antioxidant molecules is required for the complete neutralization of free radicals. The excess accumulation of free radicals provokes cellular damage and can cause several fatal diseases, including cancer, diabetes, Alzheimer’s disease, and aging [16,17]. SMs, such as polyphenols, have great potential in neutralizing free radicals, and are excellent antioxidants molecules [17]. The polyphenols derived from plant crude was shown to neutralize ROS free radicals [18]. Polyphenols scavenge the singlet and triplet oxygen-generated free radicals to provide hydrogen as a donor molecule [16]. Several studies have proved that crude extracts of different medicinal plants have high antioxidant and antimicrobial potential.

Crude extract of flowers *Wendlandia thyrsoidea*, *Olea dioica*, *Lagerstroemia speciosa,* and *Bombax malabaricum* species showed the great potential of antioxidant and antimicrobial activity, in regard to the presence of phenolic and flavonoids [19]. *Ziziphus lotus* and *Ziziphus mauritiana* leave fruit and seed extract with higher phenolic flavonoids and tannins, which show tremendous antioxidant capacity and have been successfully used against different bacterial strains [20]. Similarly, stem bark extract of *Crateva religiosa* showed antimicrobial and antifungal activity due to the presence of phenolic phytochemicals. Therefore, it can be formulated for drug discovery in the future for pharmaceutical industries [21]. Natural bioactive compounds present in *Nepeta trachonitica* showed high phenolic content, and antimicrobial as well as antioxidant activity. HPLC-MS/MS data reveal that these medicinal plants have high phenolic compounds and could be a promising source of nutraceutical and drug industries [22]. Colorimetric, chromatographic, and spectrophotometric assays revealed that *P. granatum* (pomegranate) leaf extract showed a high content of total phenols, ortho-diphenols, tannins, and antioxidant capacity, making pomegranate leaf extract a valued plant source of accepted bioactive molecules for emerging beneficial food–pharma ingredients [23].

SMs are highly economically valuable products because of the current clinical use of drug plants. They have been used extensively as a drug, flavors, fragrances, etc. Plants synthesize a considerable number of phenols and derivatives as aromatic substances [24]. Thousands of terpenoids are used extensively to produce drugs synthesized from the five-carbon precursor isopentyl diphosphate. However, around 12,000 alkaloids with nitrogen atoms are biosynthesized from amino acids. Alkaloids are used as salts in medicine, such as quinine, vinblastine, and reserpine [25,26]. Currently, alkaloids are used for analgesics, anti-cancer agents, muscle relaxants, antibiotics, and sedatives.

Furthermore, around 8000 phenolic compounds are synthesized from the malonate/acetate or shikimic acid pathway [27]. Studies reveal that phenols have antimicrobial, antiviral, and anti-inflammatory actions [28,29,30]. During oxidative damage, phenolic compounds act as antioxidants and protect against the damage of cells from oxidative stress. Phenolic compounds have neuroprotective, fungicidal, and bactericidal activities [31,32,33]. Moreover, it has been well documented that phenolic compounds have anti-atherosclerosis and anti-cancer activity [34,35].

Plant products are virtual repositories for the development of new drugs, with minimal side effects on humans. The extensive array of phytochemicals possessing antioxidant activities is are required for the therapeutic activity of plant products against human diseases, including tuberculosis [36,37]. The aqueous and ethanolic extract of *Piper sarmentosum* harboring antioxidant activity is reported to exhibit antitubercular activity [38]. The antioxidant activity determined for different parts of the selected plant using DPPH and beta-carotene linoleic acid assay displayed substantial variations. Alcoholic extract was observed to have better antioxidant potential in comparison to aqueous extract. Recently, essential oil, and a major component, viridiflorol, derived from *Allophylus edulis,* have demonstrated antioxidant and anti-tuberculosis activity [39]. The investigation used the DPPH and ABTS assay to measure antioxidant activity of significant components and essential oils. The radical scavenging activity percentage of essential oil and viridiflorol as measured by ABTS was 44.33% and 57.55%, respectively. The antioxidant value determined by the DPPH assay, and represented as IC_50_, was 82.9% and 74.7%, respectively, suggesting moderate activity compared to the reference materials (butylated hydroxytoluene and ascorbic acid). The antioxidant activity antitubercular activities of plants, including *Globularia alypum*, *Acacia catechu*, *Ailanthus excelsa*, *Aegle marmelos*, *Andrographis paniculata*, *Datura metel*, and *Aegiceras corniculatum*, have also been registered by different researchers globally [40,41,42], indicating the potential opportunities of huge plant diversity in treating life-threatening diseases (e.g., tuberculosis). However, the antioxidant activity of plants varies considerably, depending on the nature of phytochemicals, the method of extraction, climatic conditions, methods of measuring antioxidant activity, and the plant parts selected. One of the major limitations of using plant products having antioxidant activity for treating tuberculosis may be the restricted synthesis of the target compound by the plant itself. However, such hurdles can be resolved to some extent by using modern genetic engineering approaches to direct the compound synthesis in the desired quantity.

Conventional methods of metabolites screening, such as high throughput screening (HTS) and virtual high throughput screening (vHTS), have been used to speed up the drug discovery for time-efficient identification of cost-effective novel and selective metabolites. However, HTS explored bulky hydrophobic metabolites poorly suited to chemical modification, requiring higher costs and time. Few vHTS success stories have been explained, identifying plant metabolites against specific virulent proteins, such as Dengue virus proteins [43]. Docking is the greatest tool of bioinformatics employed to determine the binding pose and binding score. Docking has been considered a “leader” in the present era, performing a range of identifications of plant metabolites to candidate leads for drug development [44]. The perfect binding of the compound provides the best scoring function that “implicates” in exploring the novel candidate complex and, hence, reduces the efforts needed in experimental work. The advancements in computational technology have “escorted” the synthesis of nature-based drugs, such as dasatinib and imatinib (approved by the FDA) [45]. Network pharmacology network procedures have increased the binding associations between ligands and their targets [46]. Docking has become an important methodological feature in computer added drug design (CADD). Docking is vital in determining the novel ligand from a medicinal plant for targeted proteins for structure-based drug designs [47]. Hence, docking will help increase crucial knowledge about the therapeutic potential of plant metabolites [48].

As per the literature review, several reports and studies show the potential of natural products as antimycobacterial agents. Mitscher and Baker [49] accounted for various plant-derived compounds as potential antitubercular agents. Gautam et al. [50] reported more than 200 plants having potent anti-tuberculosis activity, signifying the potential of natural products to remedy life-threatening diseases, such as TB. Drug discoveries based on computational approaches provide novel alternative tools to reduce the expensive and tedious identification of potential drug leads. Ligand-based computational screening has been used to characterize and identify new potential inhibitors and drug repurposing [44]. Miryalaa et al. [51] worked on 15 natural compounds to explore their anti-TB properties, employing in silico methods, and compared their potential with conventional drugs against TB and their respective protein targets. Interactive studies showed that glycyrrhizin, swertiamarin, and laccaic acid exhibit better binding affinity than conventional anti-TB drugs. Hence, glycyrrhizin, laccaic acid, and swertiamarin could be used to develop multi-target alternative drug candidates. Inhibition of important enzymes responsible for vital cellular functions, hence survivability of mycobacteria in the host system, is just one critical strategy used to deal with the (continuously rising) global TB incidents. In the present study, five plant secondary metabolites (alliin, aloin, octyl-β-d-glucopyranoside, oleanolic acid, and phytol) were evaluated against two standard front line anti-TB drugs, isoniazid (ISN) and ethambutol (EMB), to decipher their potential anti-tuberculosis efficacy, targeting four of the mycobacterial receptor proteins/enzymes (arabinosyltransferase C, protein kinase A, glutamine synthetase, and proteasomal ATPase) via in silico approaches.

## 3. Current Status and Severity of Tuberculosis

Geographical data over the TB epidemic showed South-East Asia as the most affected part of the world, with 44% of the total cases alone in this region. Further, eight countries accounted for two-thirds of the global total: India (26%), Indonesia (8.5%), China (8.4%), the Philippines (6.0%), Pakistan (5.7%), Nigeria (4.4%), Bangladesh (3.6%), and South Africa (3.6%). The other 22 other countries on WHO’s list of 30 high TB-burdened countries accounted for 21% of the global total (Global Tuberculosis Report 2020 (released on 14 October 2020; https://www.who.int/publications/i/item/9789240013131). In addition, on the list of drug-resistant TB countries, India again handled the largest disease burden (130,000 new cases in 2018) in sharing with countries, i.e., China and the Russian Federation. TB statistics in India reveal the concerning burden status. There were 449,000 deaths caused by in 2018, including 2.16% of deaths of people with HIV. Among the causes of deaths in all age groups, TB was in the top five. In accordance with the previous year’s data (2000–2018), the disease incidence and disease-death rates declined in India because of the success of various treatment programs. In these TB treatment programs, the prescribed medicines cover 81% of the treatment success rate.

Nevertheless, these figures are not enough to provide relief because of the emergence of the toxic effects of synthetic drugs. Furthermore, there seems to be an increasing trend of side effect reports regarding the drug regimens administered to treat TB [52,53]. Therefore, there is an urgent need to search for safer alternatives for the treatment of TB, so that patient safety can be ensured.

## 4. Management of MDR-Mtb: A Herbal Approach

Researchers are exploring novel antimycobacterial compounds that have lesser side effects due to the development of multidrug-resistant TB and severe side effects of the synthetic drugs used for treatment. A list of side effects caused by various synthetic drugs is described in Table 1, along with generic names of the medicine.

Various plants and their metabolites elicit the desired effects against the virulent disease factors under in vivo and in vitro conditions. Plant-derived chemicals proved to be the better mycobacteria-inhibitory substances, with less (or no) side effects, ensuring the fast recovery of the patients. Jimenez-Arellanes et al. [58] evaluated the antimycotic activity of aqueous, methanolic, and n-hexane extract of 22 different plants against *M. tuberculosis* H37Rv and *M. avium* at concentrations ranging from 50 to 200 µg/mL. In a case study, Fauziyah et al. [59] checked the efficiency of the combined effects of anti-tuberculosis drugs and ethanolic extract of some specific medicinal plants against multi-drug resistant Mtb isolates. They concluded that a combination of plant extracts and rifampicin achieved better effects against the rifampicin/streptomycin-resistant strain. However, they also observed the antagonistic effects with streptomycin, ethambutol, and isoniazid. Nowadays, plant extracts and their metabolites are broadly used to treat MDR in several other human pathogens, viz. *Staphylococcus aureus* (wound and bloodstream infections), Escherichia coli (causing urinary tract infections), and Klebsiella pneumoniae (causing pneumonia, urinary tract, and bloodstream infections). It is estimated that between 2005 and 2015, a total of 110 purified compounds and 60 plant extracts were obtained from 112 different plants having potential effectiveness against MDR pathogens [60]. Details of the plants showing antimycobacterial activity are listed in Table 2.

## 5. Computational Analysis

### 5.1. Selection and Retrieval of Receptor Proteins

The selection procedures of receptor proteins were purely based on the literature survey. To assess the multi-domain antimycobacterial activity of ligands, different types of receptor proteins (mycobacterial proteins) were taken into consideration that had different 3D structures and had different functions as well; specifically, they all must have had some critical functions required for the survival of target bacterial cell and its infection. The selected receptor proteins arabinosyltransferase C (PDB ID: 3PTY), protein kinase A (PDB ID: 4OW8), glutamine synthetase (PDB ID: 3ZXR), and proteasomal ATPase (PDB ID: 5KWA) all have some critical functions to perform the vital cellular functions. The arabinosyltransferase C, belonging to enzyme class transferase, is a vital enzyme, playing a pivotal role in critical biological processes, and it participates in the biosynthesis of the essential part of the *Mycobacterium* cell wall [93]. Protein kinase A is recognized for its significant contribution in regulating the *Mycobacterium* cell shape and its mechanics. This protein gets exponentially upregulated during mycobacterial growth and infections [94]. Mycobacterial glutamine synthetase is known to increase the bacterium capacity to inhibit the host’s phagosome–lysosome defense mechanism. It is also actively involved in cell wall biosynthesis and in converting glutamate, ammonia, and ATP to glutamine, phosphate, and ADP in bacterial cells [95]. At the same time, proteasomal ATPase from the bacterium is an essential virulent factor required for infection in humans [96]. Searching for a multi-domain mycobacterial inhibitory molecule, using all of the aforementioned receptor proteins, will aid in the discovery of novel inhibitory compounds with an overall inhibitory effect on the target bacterium.

**Figure 1:**
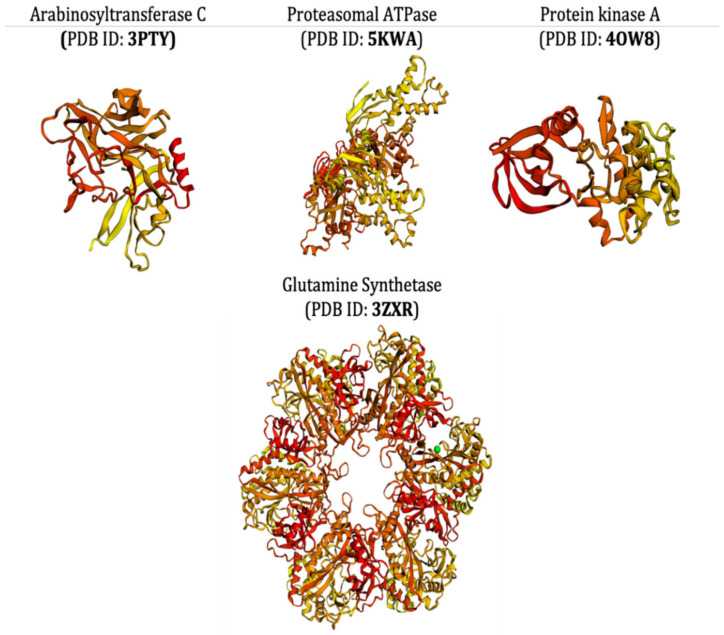
The 3D structure of the PDB-retrieved receptor proteins of *M. tuberculosis*.

The crystal structures of receptor proteins were procured in the form of atomic coordinates from the Protein Data Bank (https://www.rcsb.org; accessed on 25 October 2021), using specific PDB IDs of each protein. The unwanted water molecules, heteroatoms, and other ligand coordinates were removed from the protein structures to obtain a more suitable and stable conformation [97]. For the docking algorithm, along with the addition of polar hydrogens, Kollman charges were added to each protein molecule and saved in pdbqt format. The 3D structure of the receptor proteins is illustrated in Figure 1.

### 5.2. Selection and Retrieval of Ligand and Molecules

Selections of the plant’s secondary metabolites (test ligands) were based on the literature survey, inferring their biological activity for the well-being of human health. Alliin (S-allyl-L-cysteine sulfoxide), the most abundant sulfur compound in *Allium sativum* L., has been reported as a potent cardioprotective and neuroprotective agent having antidiabetic, anticholesteremic, and anticarcinogenic effects [98,99]. *Aloe vera* is a medicinal plant that exerts a hypoglycemic effect with no side effects [100]. Aloin, a major compound of *A. vera* latex, is a well-known laxative agent, generally existing as a mixture of two diastereoisomers, aloin A and aloin B, also referred to as barbaloin and isobarbaloin, respectively [101]. *Phyllanthus emblica*, commonly known as ‘amLa’ in India, has been used for treating various human ailments for centuries. The major bioactive compounds of *P. emblica,* including octyl-β-D-glucopyranoside, have been well known for curing effects against varied diseases, such as fever, cough, piles, constipation, anorexia, hemorrhoids, skin diseases, asthma, biliousness, respiratory disorders, tumors, and cancer [102]. Phillips et al. [103] explained that the oleanolic acid isolated from *Lantana hispida* showed a potential inhibitory potential against *M. tuberculosis* H37RV (Table 2). Still, its inhibitory mechanism of action (MOA) is unknown; therefore, assessing its inhibitory activities against selected receptor proteins will give insight into its working MOA. (E)-phytol, a metabolite from *Leucas volkensii,* was also reported to have inhibitory activity against *M. tuberculosis* H37Rv (MIC: 2 μg/mL) and is believed to be a better therapeutic agent for the treatment of TB [71,104,105].

The control ligands, i.e., isoniazid (ISN) and ethambutol (EMB), are used predominantly in the treatment of TB. However, they are known to cause severe side effects on patients and are failing in inhibitory termination of the *Mycobacterium*. Therefore, novel antimycobacterial molecules harboring less (or no) side effects are currently being searched for, and are highly desirable in current incidents of TB.

**Figure 2:**
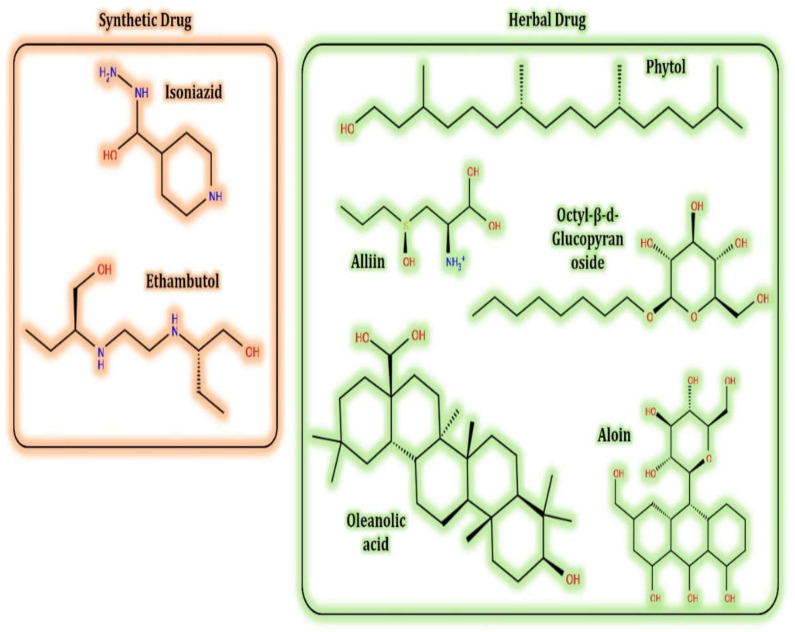
The 2D structure of the secondary metabolites from diverse plants.

The native structures of all the ligand molecules were fetched from the PubChem database (https://pubchem.ncbi.nlm.nih.gov; accessed on 25 October 2021) using Chimera software and saved in pdbqt format. The 2D structures of all the ligands are presented in Figure 2.

### 5.3. Docking Algorithm

For the docking algorithm calculations, AutoDock 4.2 package (http://autodock.scripps.edu/resources/adt; accessed on 25 October 2021) was used. The ligand’s native structures were blindly docked in the potential binding cavities of the receptor proteins. The AutoDock package executes the binding predictions of the ligands vs. receptor molecules based on the empirical force field and the Lamarckian genetic algorithm. The binding energy persuaded the binding affinity between the target sites and functional groups of ligand molecules utilizing various interactions viz. H-bonds, ionic interactions, hydrophobic interactions, and van der Waal’s forces. With these understandings, the visual analysis of the docking algorithm executed between the selected ligands and receptor proteins is shown in Table 3. Chimera software was used to visualize the binding cascade of the ligand against the receptor protein complex.

**Figure 3:**
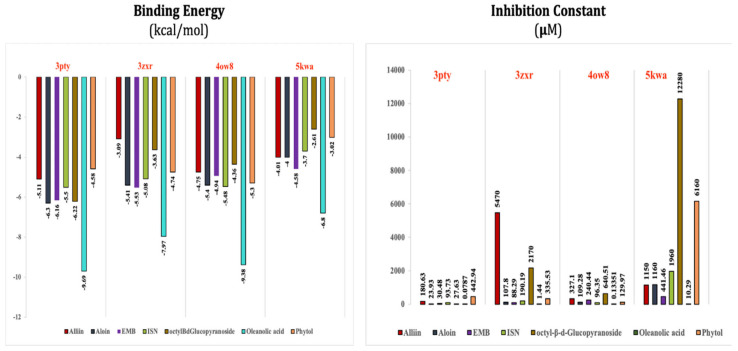
The binding energy and inhibitory constant of phytomolecules obtained after complexation with receptor proteins of *M. tuberculosis* (details also described in Supplementary Table S1).

The binding energy and inhibitory constant of ligands obtained against their interactions with receptor proteins are presented in Figure 3.

Furthermore, more details regarding the interactive venture of ligands with active sites of the receptor protein can be seen in Table 4.

Among all of the test ligands analyzed, oleanolic acid performed best in multi-regimen inhibition of *Mycobacterium*. It showed efficient binding, with all four receptor proteins having the most negative binding energies among all ligand–receptor interactions. Energetically, oleanolic acid vs. arabinosyltransferase C was the most favored interaction, having a binding energy of −9.69 kcal/mol. Other test ligands viz. alliin, aloin, octyl-β-D-glucopyranoside, and (E)-phytol showed similar affinity towards the receptor proteins as the control ligands, ISN and EMB. Despite exhibiting antimycobacterial activity, (E)-phytol did not perform as expected against the selected receptor proteins. However, all of the test ligands presented efficient multi-domain inhibitory functions against the Mtb.

The inhibition constant value (Ki) of all ligand–receptor interactions indicates the inhibitory potential of the ligands against the selected receptor protein. The Ki value represents the ligand concentration required to inhibit the activity of half of the amount of receptor proteins. It is inversely proportional to the binding affinity of the ligands. Oleanolic acid showed the lowest possible Ki against all of the receptor proteins among the inhibitory potential of the ligands. In the oleanolic acid vs. arabinosyltransferase C interaction complex, the lowest Ki value, i.e., 0.0787 µM, was observed. All other test ligands, except octyl-β-D-glucopyranoside, performed similarly with the control ligands.

### 5.4. Toxicity Assay of Test Ligands

The cytotoxicity of test ligands was assessed via the in silico web facility ProTox-II (http://tox.charite.de/protox_II; accessed on 25 October 2021) and found to be ineffective against humans.

## 6. Bioinformatics Opportunities for Medicinal Plant Studies

Plants have been used as therapeutic regimens since immemorial periods, and various commercially significant medicines are derived from plants. However, traditional methods used to explore plant-based regimens are timely and are highly expensive. Moreover, such extensive works have faced several problems in keeping up with the hasty advancement of high throughput technologies. In this era of high volume, high-throughput data production in life sciences—bioinformatics plays an essential role in overcoming the above-mentioned problems, with limited time and expenditure in drug design and discovery [106,107].

Nowadays, bioinformatics plays a crucial role in exploring the role of medicinal plants against various diseases, diabetes, cancer, and tuberculosis. With ever-increasing genomic and proteomic studies, it is essential to decipher the data competently. Bioinformatics plays a crucial role in exploring new genetic factors, driving the identification of several new genes and proteins. In addition, its tools have aided in explaining significant relationships between several molecular factors [108]. Thus, bioinformatic approaches, such as molecular docking, RMSD value, etc., help in the screening of plant metabolites, to develop drugs that target virulent factors associated with molecular pathways.

## 7. Concluding Remarks and Future Directions

TB is a severe disease; its treatment started 73 years after the discovery of streptomycin and other drugs. TB is also one of the world’s top- ten infectious diseases. The emergence of MDR/XDR/TDR-Mtb strains has worsened the situation, causing a severe threat to human health. The present research claims that chemotherapy using synthetic anti-tuberculosis drugs is not very efficient at killing the dormant and intracellular forms of Mtb. Researchers are focusing on secondary metabolites in plants due to their therapeutic potential. However, there is still a need to resolve some concerns, for better application of secondary metabolites and to effectively manage human diseases (including tuberculosis). Major concerns include (a) lower animal experimentation facilities used for investigating the in vivo effects of phytomolecules; (b) lower solubility of the natural products; and (c) unavailability of sufficient amounts of pure compounds. Considering the above, there is a need to identify more anti-tuberculosis phytomolecules that have selective neutralizing activities against specific target proteins, using computational and bioinformatics approaches (Figure 3). The application of improved extraction procedures, high throughput techniques for precise determination of bioactive molecules, and structural elucidation of novel chemical molecules expressing potential effectiveness against different clinical isolates and isolates with MDR/XDR/TDR characteristics, is highly imperative. The extensive investigation of in vitro mechanisms of action of molecules showing better outcomes under in silico studies could help design molecules and analogs with minimal side effects. Information pertaining to important proteins participating in the survival of mycobacteria in the host system and a complete understanding of biosynthetic pathways and genetic regulation might facilitate the development of natural products with considerable inhibitory action against *Mycobacterium tuberculosis* and other species. In addition, the in vitro cytotoxicity of plant metabolites should be considered while searching for new candidate molecules with potential anti-tuberculosis effects. Such studies would not only solve the disease burden of TB by identifying the novel structures of variant compounds against resistant and non-resistant Mtb strains, but would also facilitate raising the importance of the therapeutic properties of medicinal plants in modern medicine.

## Figures and Tables

**Table 1:** The list of side effects caused by various synthetic drugs, along with generic names.

Generic Names	Medicinal Compound	Side Effects	Mode of Action	References
Isoniazid, isoniazide, azuren, INH, L 1945, Mybasan, neumadin, RP 5015, tubomel, vazadrine, isoniazidum	Isonicotinic acid hydrazide (isoniazid)	Hepatotoxic (hepatitis, nausea, vomiting, and decreased appetite)	Suppresses the multiplication of mycobacteria	[54]
Streptomicina, streptomycin, streptomycine, strepidin-4-α-streptobiosaminosid, streptomycin sulfate, streptomycini sulfas, streptomycinsulfat	Streptomycin	Ototoxicity	Inhibition of protein synthesis of mycobacteria in the ribosome	[55]
Ethionamide, TH 1314, aethionamidum, Bayer 5312, etionizina, ETP, ethionamidum	Ethionamide	Hepatitis, depression, hypersensitivity	A prodrug that is activated by the enzyme ethA, a mono-oxygenase in *Mycobacterium tuberculosis*; binds NAD+ to form an adduct that inhibits InhA in the same way as isoniazid	
Cycloserine, lilly 106-7, MK 65, PA 94, Ro 1-9213, SC 49088, cicloserina, cycloserinum	Cycloserine	Psychosis, rashes	Cycloserine is a broad-spectrum antibiotic with only moderate anti-TB activity. It inhibits cell wall synthesis. The MIC of cycloserine in the Bactec 460-TB system is 25–75 μg/mL	
Capreomycin sulfate, capreomycin, CAM, capromycin, L 29275	Capreomycin	Deafness, vestibular toxicity	Inhibit protein synthesis by binding to the 70S ribosomal unit	
Kanamicina, kanamycin, kanamycine, kanamycin monosulfate, kanamycin sulfate, kanamycin acid sulfate, kanamycin monosulfate, kanamycinmonosulfat	Kanamycin	Deafness, nephrotoxic	Inhibits protein synthesis by tightly binding to the conserved A site of 16S rRNA in the 30S ribosomal subunit	
Rifampicin	Rifampicin	Hepatotoxic, interaction with other drugs, a potent inducer of microsomal enzymes	Inhibits bacterial DNA-dependent RNA synthesis by inhibiting bacterial DNA-dependent RNA polymerase	[56]
Pirazinamide, pyrazinamide, pyrazinecarboxamide, pyrazinoic acid amide, pyrizinamide, pyrazinamidum	Pyrazinamide	Hepatitis, Hyperuricemia, arthralgia, arthritis	It diffuses into the granuloma of *M. tuberculosis*, where the tuberculosis enzyme pyrazinamidase converts pyrazinamide to the active form of pyrazinoic acid	
Ethambutol, ethambutolo, ethambutol hydrochloride, CL 40881, ethambutol hydrochloride, ethambutoldihydrochlorid, ethambutoli hydrochloridum	Ethambutol	Optic neuritis	It works by obstructing the formation of the cell wall. Mycolic acids attach to the 5’-hydroxyl groups of D-arabinose residues of arabinogalactan and form mycolylarabinogalactan-peptidoglycan complex in the cell wall	[57]
Protionamide, PTH, PTP, RP, protionamidum, prothionamide	Prothionamide	Hepatotoxic, hypersensitivity, idiosyncrasy	It is activated by mono-oxygenase (EthA), forms covalent adducts with nicotinamide adenine dinucleotide (NAD), and inhibits InhA, leading to blocking of the mycolic acid pathway	
P.A.S., Para-aminosalicylic acid, pasalicylum, aminosalicylic acid, aminosalicylate sodium, para-aminosalicylsaures natrium-2-wasser, parasal sodium, sodium para-aminosalicylate, natrii aminosalicylas dihydricus, sodium aminosalicylate dihydrate	Para-aminosalicylic acid	Hepatotoxic, hypersensitivity, idiosyncrasy	It targets dihydrofolate reductase (DHFR); it is incorporated into the folate pathway by two enzymes, dihydropteroate synthase (DHPS) and dihydrofolate synthase (DHFS) to produce a hydroxyl dihydrofolate compound that inhibits DHFR, and subsequently blocks the folate pathway	

**Table 2:** List of reported plant extracts with anti-tuberculosis activities against different *M. tuberculosis* isolates.

Plant (Bioactive Compound)	Extract	Mtb	MIC	References
*Lantana hispida* (-acetoxy-22-(2′-methyl-2Z-butenyloxy)-12-oleanen-28-oic acid, hydroxy-22β-(2′-methyl-2Z-butenoyloxy)-12-oleanen-28-oic acid and oleanolic acid)	Hexane extract	*Mycobacterium tuberculosis* strain H37Rv	50, 50 and 25 μg/mL respectively	[61]
*Taxus baccata*	Chloroform extract of heartwood and ethanolic extract of leaves	*M. tuberculosis* strain H37Ra	200 μg/mL	[62]
*Adhatoda vasica* (2-acetyl benzylamine and vasicine acetate)	Hexane extract	Mtb	200 and 50 μg/mL, respectively	[63]
*Terminalia phanerophlebia*	Ethanolic extract of leaves	*M. tuberculosis* H37Ra	390 μg/mL	[64]
*Opuntia ficus-indica*	Methanolic extract of the plant (summer season)	*M. tuberculosis* strain H37Rv (ATCC 27294)	50 μg/mL	[65]
*Angiopteris evecta*	Methanolic extract of leaves	*M. tuberculosis* H37Rv	400 μg/mL	[66]
*Costus speciosus, Piper sarmentosum, Pluchea indica, Pluchea indica,* and *Tabernaemontana coronaria*	Methanolic extract		800 μg/mL	
*Zanthoxylum capense* (Decarine)	Methanolic extract of roots	*M. tuberculosis* H37Ra (ATCC 25177) and *M. tuberculosis* H37Rv (ATCC 27294)	1.6 μg/mL	[67]
*Helichrysum devium*	Methanolic extract	*M. tuberculosis* H37Rv	50 μg/mL	[68]
*H. melaleucum*			100 μg/mL	
*H. obconicum*			200 μg/mL	
*Artemisia capillaris* (hydroquinone and ursolic acid)	Methanolic extract	*M. tuberculosis* strain H37Rv and two clinical isolates (resistant and sensitive)	12.5 μg/mL against sensitive strains of Mtb while a range of 12.5 to 25 μg/mL against the resistant strains	[69]
*Curtisia dentata*	Methanolic extract of leaves	*M. tuberculosis* H37RV (ATCC 27294)	22.2 μg/mL	[70]
*Curtisia dentata* (ursolic acid acetate)	Ethanolic extract		3.4 µg/mL	
*Aristolochia taliscana* (Licarin A)	Hexane extract	*M. tuberculosis* strains: H37Rv, four mono-resistant H37Rv variants and 12 clinical MDR isolates	3.12–12.5 μg/mL	[71]
*Excoecaria agallocha*	Methanolic extract	*M. tuberculosis* H37Rv and two clinical isolates of Mtb	88.95% of antimycobacterial activity against *M. tuberculosis* H37Rv while 70.02% and 82.54% for other two isolates at 500 µg/mL concentration	[72]
*Lantana camara*	Chloroform and methanol extracts of leaves	*Mycobacterium tuberculosis* H37Rv, rifampicin-resistant TMC-331 and a non-resistant wildstrain (28–25271	5.0 mg/mL to 50.0 mg/mL	[73]
*Solanum torvum* Sw.	hydro-ethanolic extracts	*Mycobacterium tuberculosis* H37Ra	156.3 µg/mL	[74]
*Alpinia galanga* L. Willd.	Acetone, aqueous and ethanolic extracts of rhizomes	*Mycobacterium tuberculosis* (M.tb) H37Rv	50–100 μg/mL	[75]
*Lantana camara* L., *Euphorbia hirta* L., *Mukia maderaspatana* (L.) *M. Roem*, and *Abutilon indicum* L.	Methanolic crude extracts	*Mycobacterium tuberculosis* (Mtb) and Mtb H37Rv	400–1600 μg/mL	[76]
*Artemisia annua*	Dichloromethane extracts	*Mycobacterium tuberculosis* (Mtb), *Mycobacterium abscessus*	37.5 µg/mL	[77]
and *A. afra*			<1.3 µg/mL	
*Zingiber officinale*	Hydroethanolic extract of rhizomes	*M. tuberculosis* H37Rv (ATCC 27294)	1250 μg/mL	[78]
*Vitellaria paradoxa*	Hydroethanolic extract of bark		78.13 μg/mL	
*Alstonia boonei*			156 μg/mL	
*Musa* spp. AAB, cv. ‘‘Manzano’’	n-hexane extract and ethyl acetate extract	*Mycobacterium tuberculosis*	12.5 and 6.25 l g/mL	[79]
*Trixis angustifolia*	Hexane extract	*Mycobacterium tuberculosis* H37Rv	12.5- 25.0 μg/mL	[80]
*Acacia farnesiana*	hexane, chloroform and methanolic extracts	*Mycobacterium tuberculosis* H37Rv and G122	100–200 µg/mL	[81]
*Pterolobium stellatum* (Forssk)	Chloroform extracts	*M. tuberculosis* strain H37RV	0.312 mg/mL	[82]
*Persea americana* Mill L.			2.5 mg/mL	
*Otostegia integrifolia* Benth L.			0.312 mg/mL	
*Aegle marmelos* L, *Glycyrrhiza glabra* L, *Lawsonia inermis* L, *Piper nigrum* L, and *Syzygium aromaticum* L.	Methanolic extract	*M. tuberculosis* strain H37RV	0.8 to 100 μg/mL	[83]
*Boswellia serrata* Roxb. ex, *Datura stramonium* L and *Lavandula stoechas* L.	Ethanolic extracts	*M. tuberculosis* strain H37RV	125 to 250 μg/mL	[84]
*Pinus merkusii*	Ethanolic extract	*Mycobacterium tuberculosis* H37Rv	1000 µg/mL	[85]
*Dendrophthoe falcata* L.	Ethanol water and methanol: water extracts	*Mycobacterium tuberculosis* (H37Rv strain)	6.25 μg/mL	[86]
*Tridax procumbens* L.			0.8 μg/mL	
*Triclisia gilletii*	Methanol extract	*Mycobacterium tuberculosis*	3.90 to 62.5 μg/mL	[87]
*Combretum hereroense*	Hexane, dichloromethane, methanol, and acetone	*M. smegmatis* (ATCC 1441), *M. tuberculosis* (ATCC H37Rv)	1.6 mg/mL and 1.3 mg/mL	[88]
*Citrus lemon*			0.3 mg/mL	
*Apodytes dimidiata*			1.3 mg/mL	
*Cinnamomum verum*	Aqueous & methanolic extracts	*Mycobacterium tuberculosis* H37Rv	10 mcg/mL	[89]
*Solanum surattense*				
*Costus speciosus, Cymbopogon citratus*, and *Tabernaemontana coronaria*	Methanol extracts	*Mycobacterium tuberculosis* H37Rv	100–200 μg/mL	[90]
*Croton tonkinensis*	Methylene chloride extracts	*M. tuberculosis* H37Ra, H37Rv	6.25 and 12.5 µg/mL	[91]
*Melia azedarach* L. and *Lobelia chinensis* Lour.	Methanol and n-hexane extract	*M. tuberculosis*	100 µg/mL	[92]

**Table 3:** Docking algorithm executed between the selected ligands and receptor proteins.

	Interaction	Ligand	Interaction
3pty			
Alliin	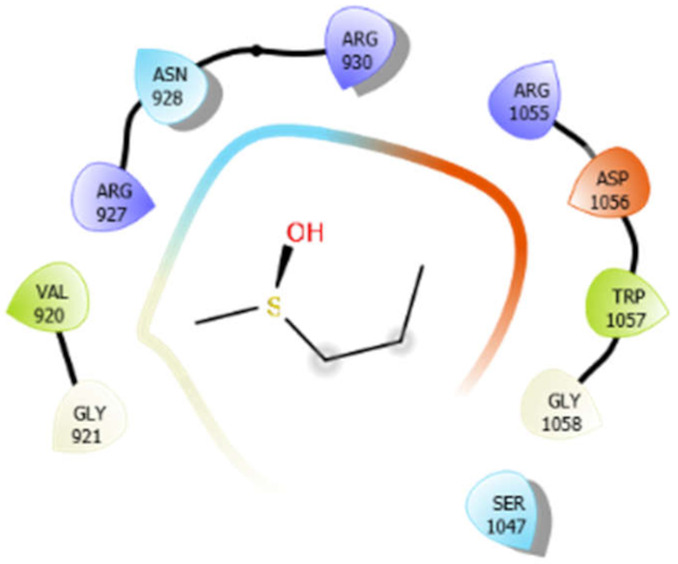	Aloin	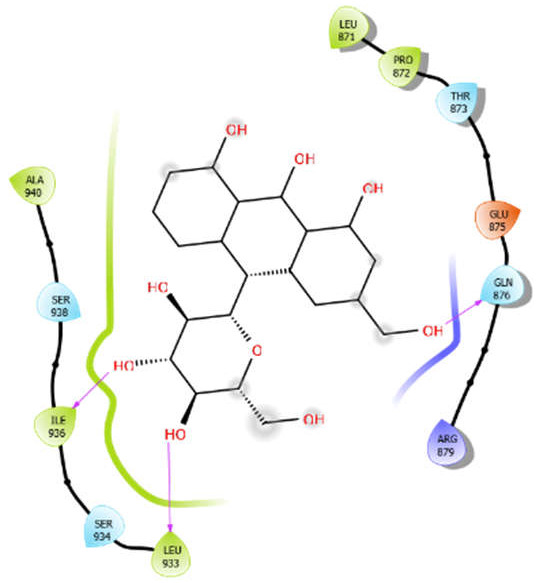
EMB	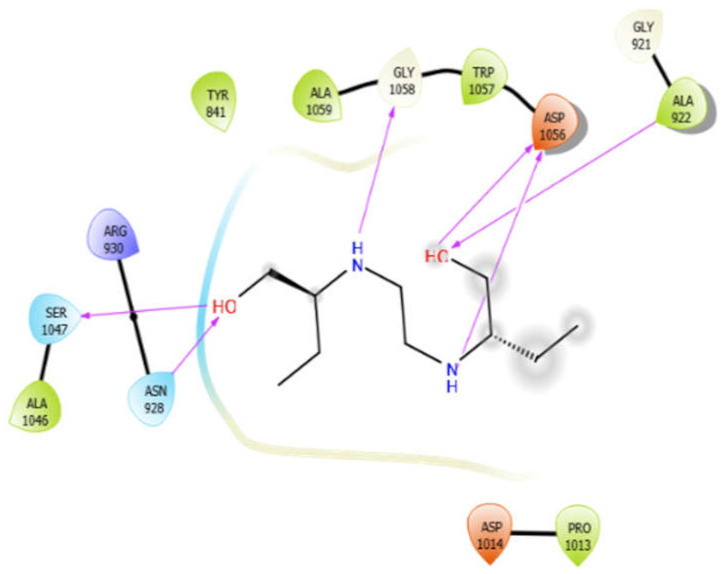	ISN	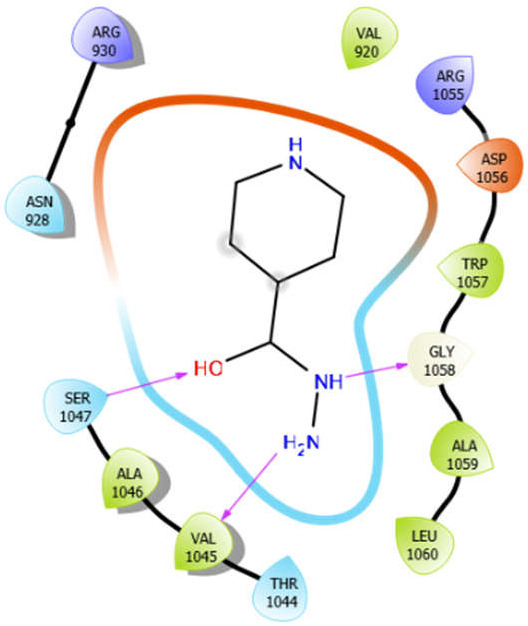
Octyl-β-d-Glucopyranoside	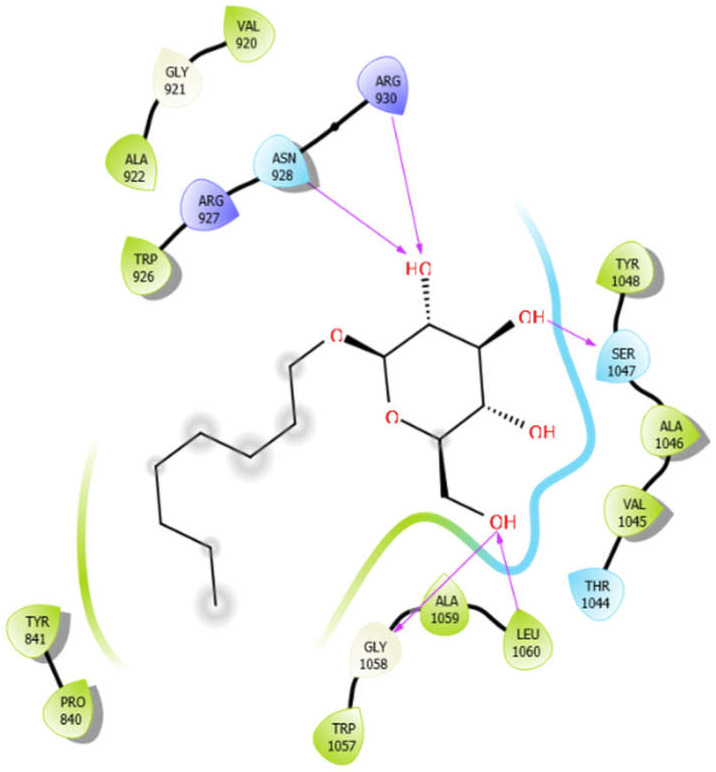	Oleanolic acid	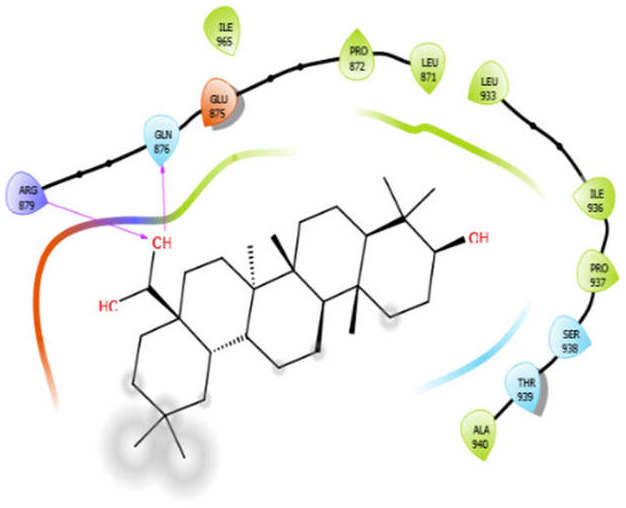
Phytol	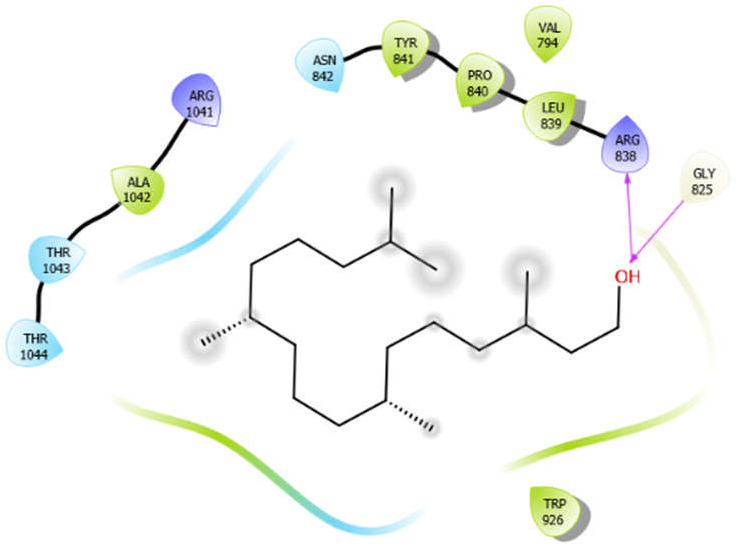		
3zxr			
Alliin	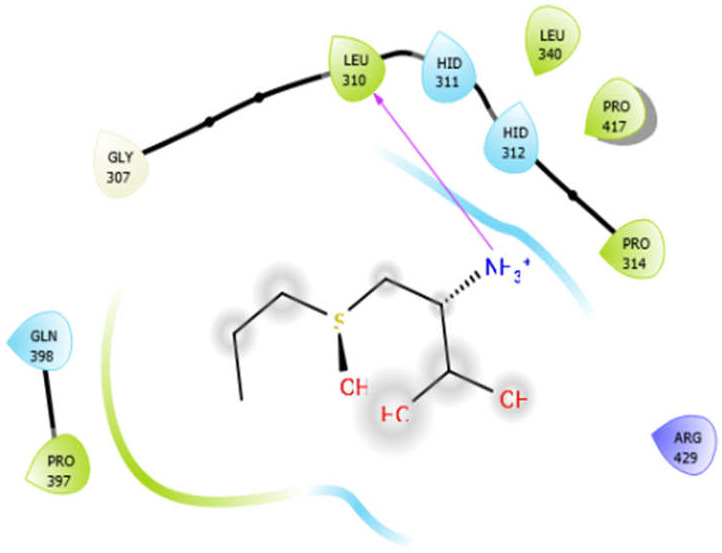	Aloin	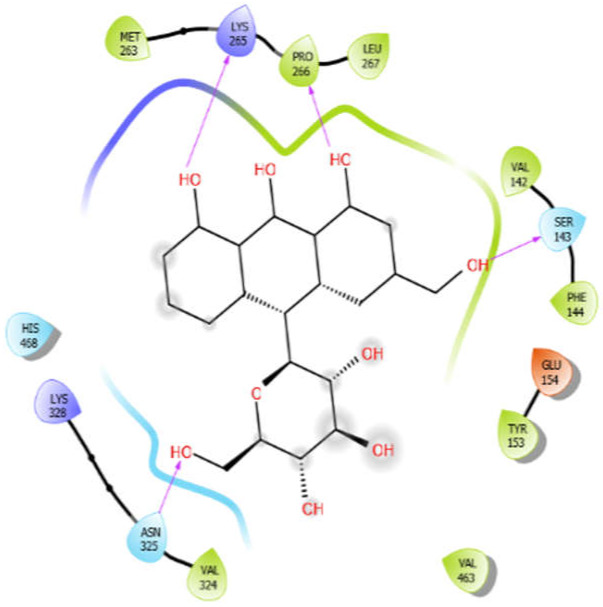
EMB	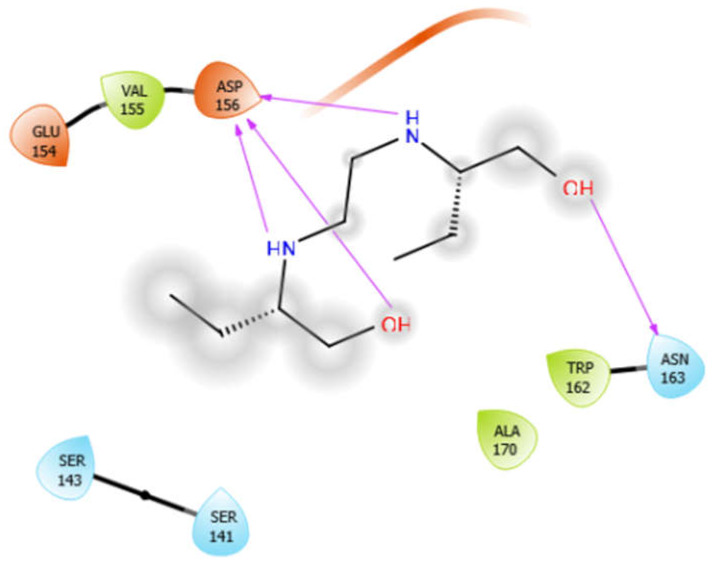	ISN	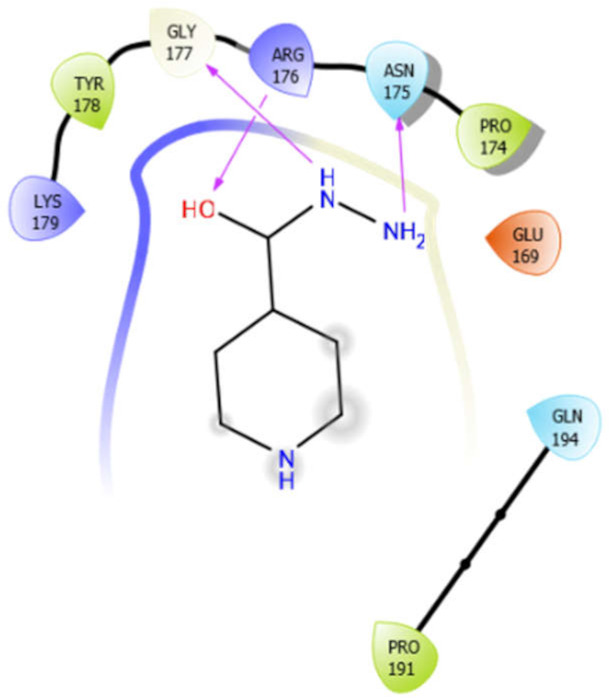
Octyl-β-d-Glucopyranoside	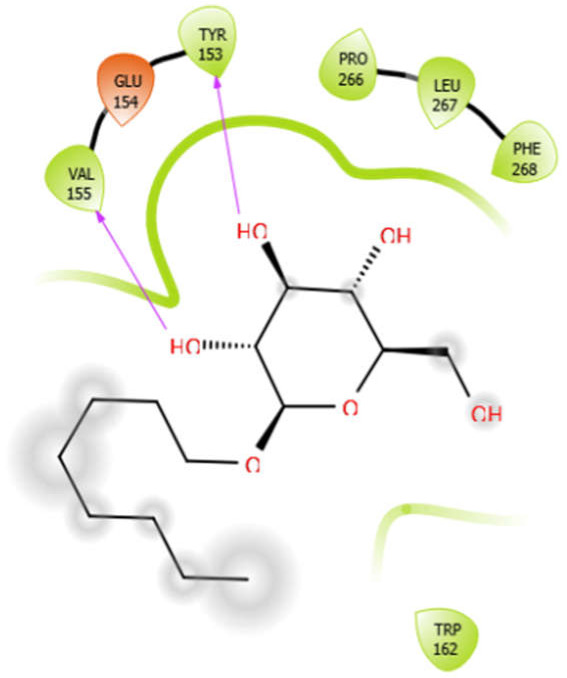	Oleanolic acid	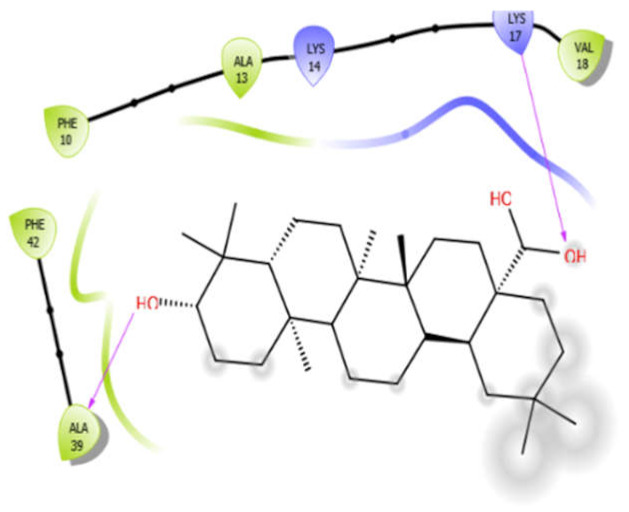
Phytol	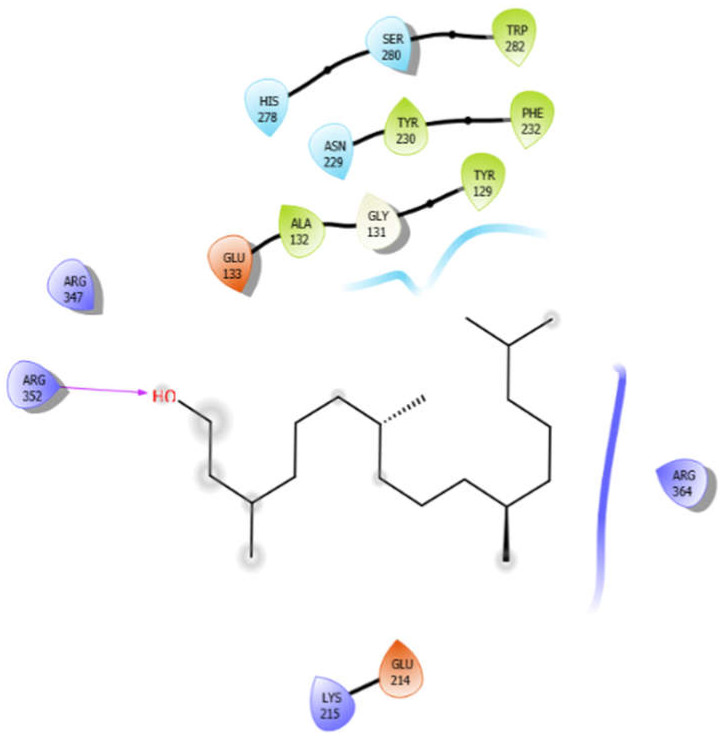		
4ow8			
Alliin	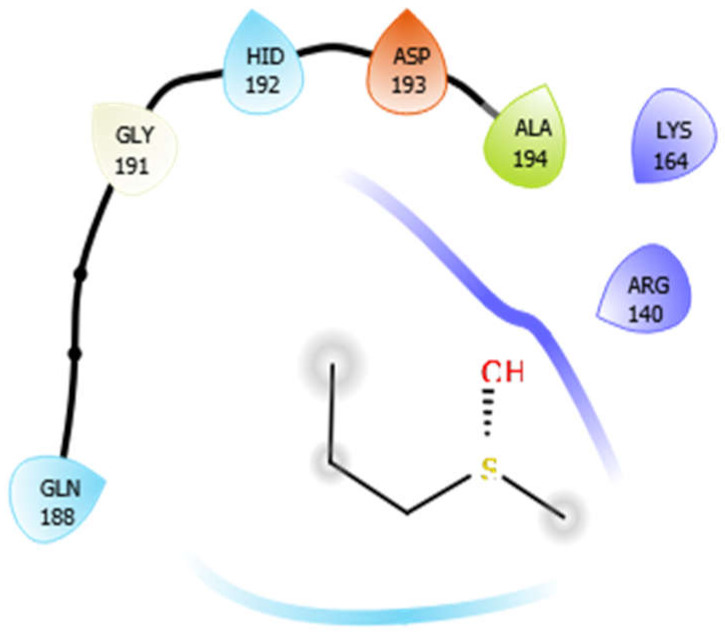	Aloin	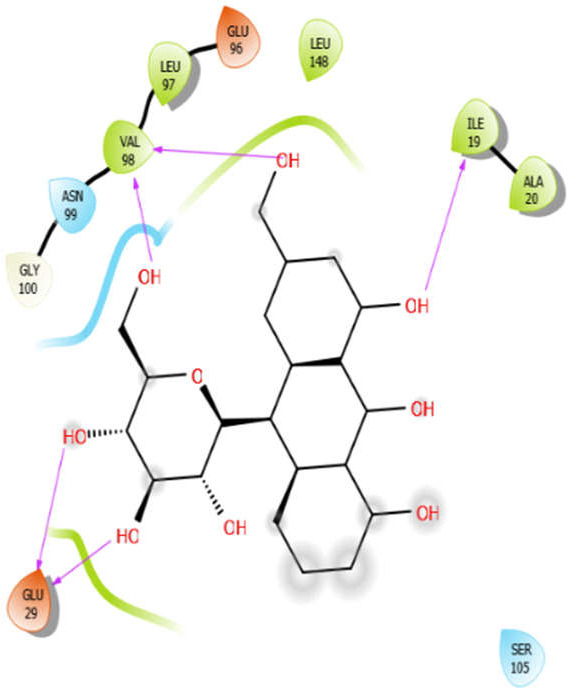
EMB	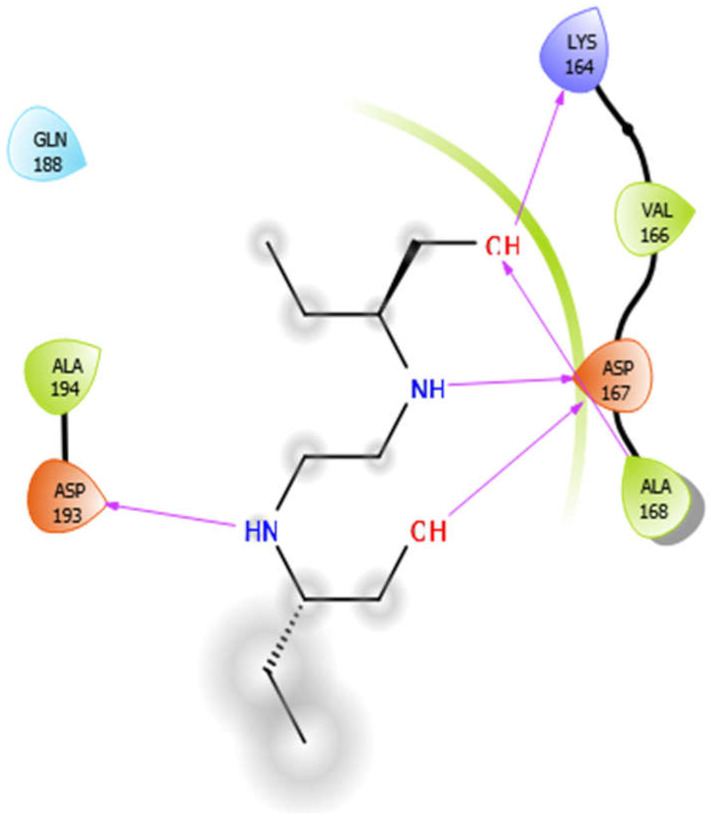	ISN	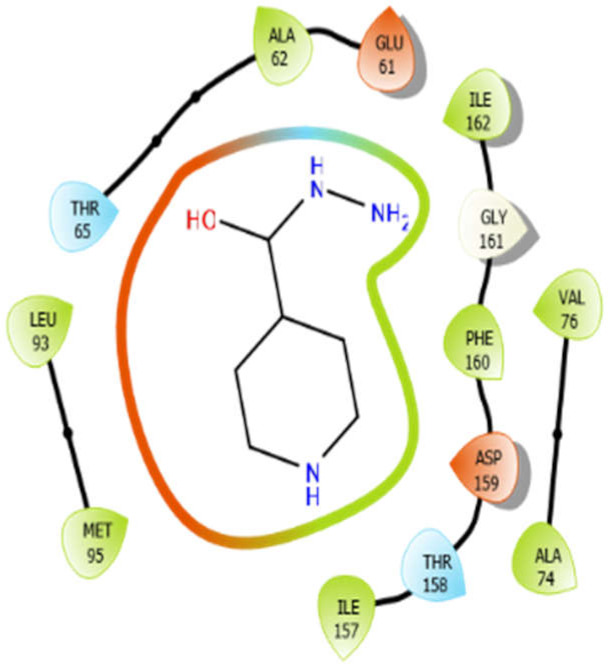
Octyl-β-d-Glucopyranoside	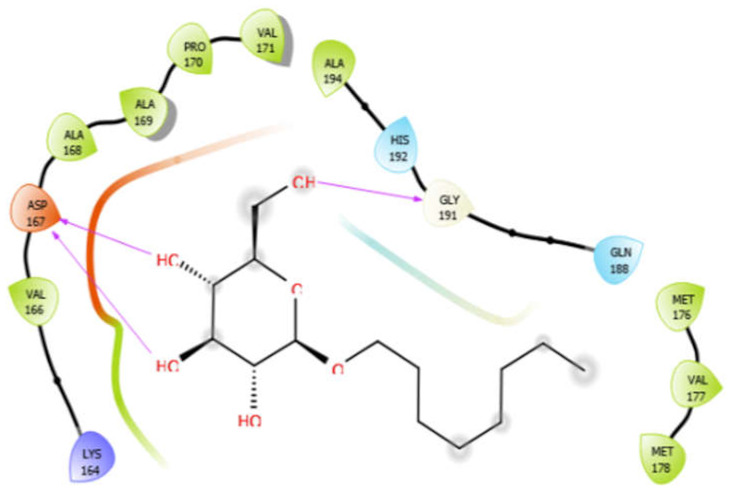	Oleanolic acid	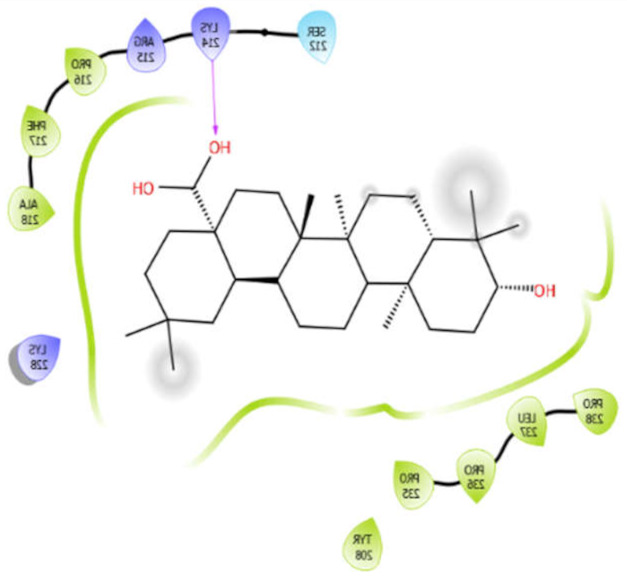
Phytol	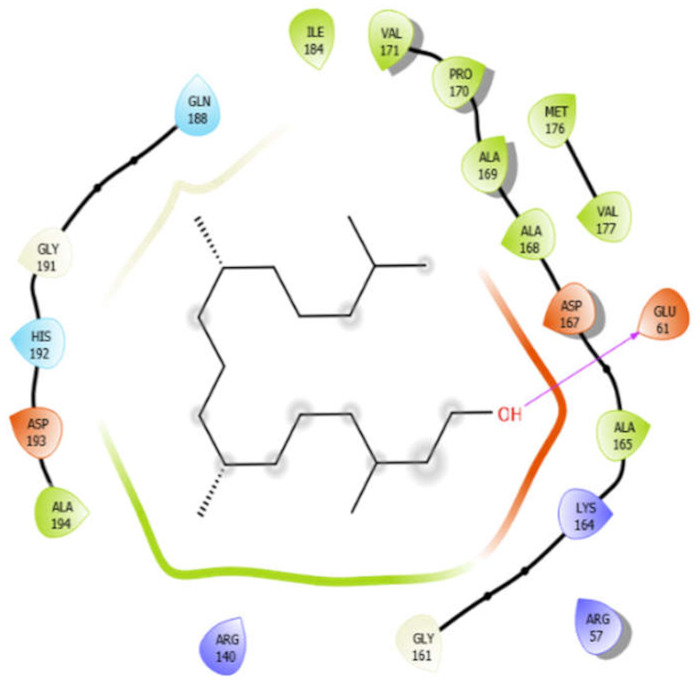		
5kwa			
Alliin	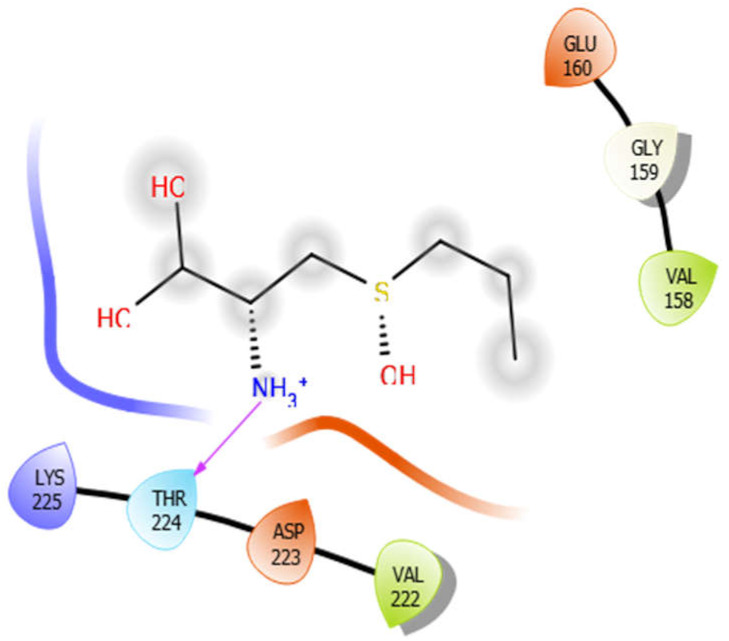	Aloin	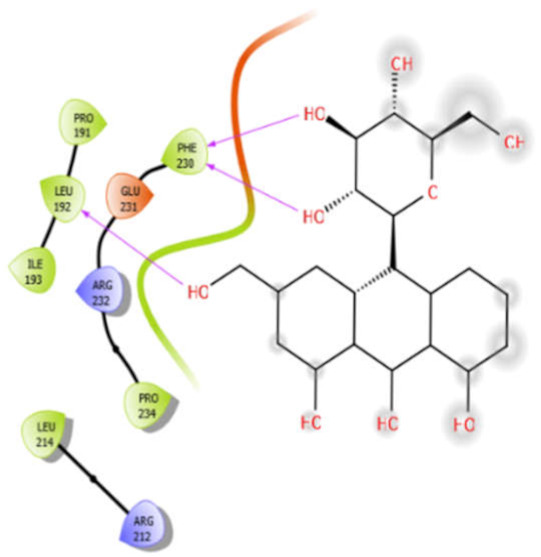
EMB	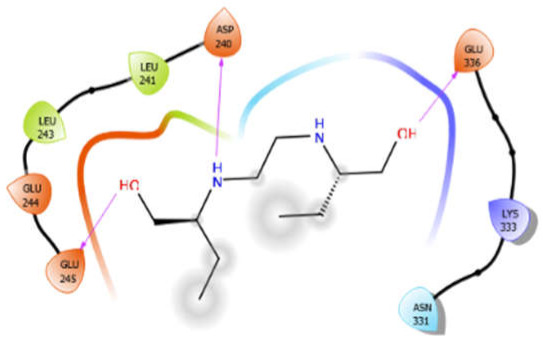	ISN	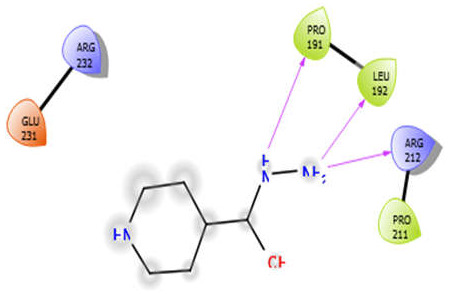
Octyl-β-d-Glucopyranoside	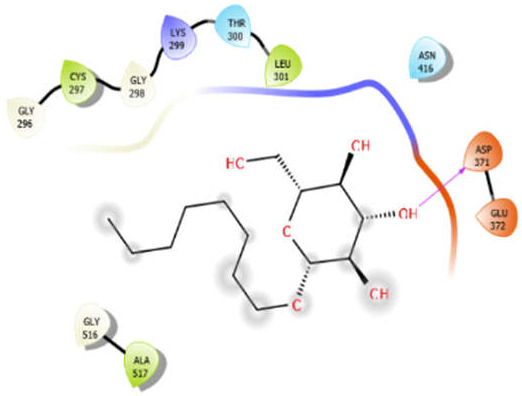	Oleanolic acid	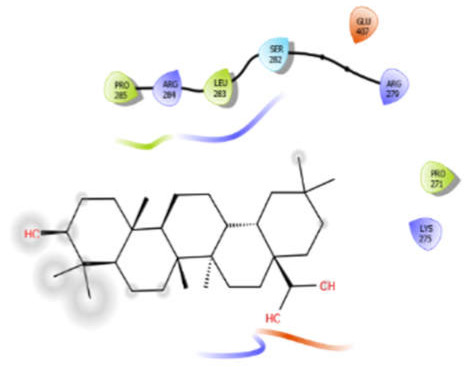
Phytol	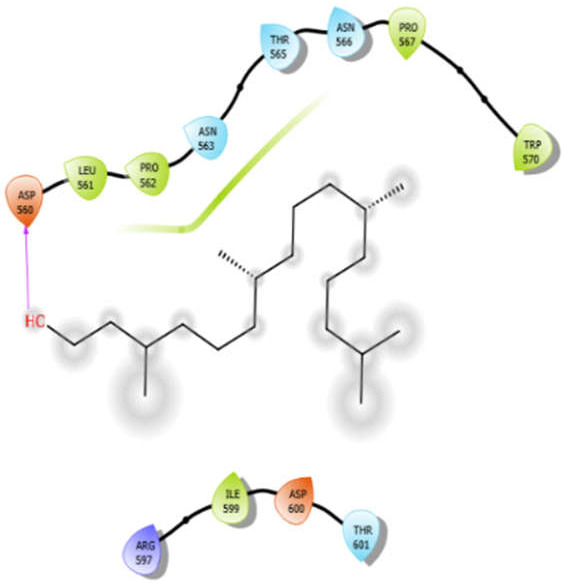		

**Table 4:** The binding energy of interacted residues of receptors proteins of *M. tuberculosis* with diverse phytomolecules.

Interactions	Bond Type	Resides and Their Legends	Binding Energy (kcal/mol)
Alliin with 3pty	Hydrogen bond	GLY921, GLY1058	−5.11
	Hydrophobic bond	VAL920, TRP1057	
	Polar bond	ASN928, SER1047	
	Charged bond	ARG927, ARG930, ARG1055, ASP1056	
Aloin with 3pty	Hydrophobic bond	LEU871, PRO872, LEU933, ILE936, ALA940	−6.3
	Polar bond	THR873, GUN876, SER934, SER938	
	Charged bond	GLU875, ARG879	
EMB with 3pty	Hydrogen bond	GLY921, GLY1058	−6.16
	Hydrophobic bond	TYR841, ALA922, PRO1013, ALA1046, TRP1057, ALA1059,	
	Polar bond	ASN928, SER1047	
	Charged bond	ARG930, ASP1014, ASP1056	
ISN with 3pty	Hydrogen bond	GLY1058	−5.5
	Hydrophobic bond	VAL920, VAL1045, ALA1046, TRP1057, ALA1059, LEU1060	
	Polar bond	ASN928, THR1044, SER1047	
	Charged bond	ARG930, ARG1055, ASP1056	
Octyl-β-d-Glucopyranoside with 3pty	Hydrogen bond	GLY921, GLY1058	−6.22
	Hydrophobic bond	PRO840, TYR841, VAL920, ALA922, TRP926, VAL1045, ALA1046, TYR1048, TRP1057, ALA1059, LEU1060	
	Polar bond	ASN928, THR1044, SER1047	
	Charged bond	ARG927, ARG930	
Oleanolic acid with 3pty	Hydrophobic bond	LEU871, PRO872, LEU933, ILE936, PRO937, ALA940, ILE965	−9.69
	Polar bond	GLN876, SER938, THR939	
	Charged bond	GLU875, ARG879	
Phytol with 3pty	Hydrogen bond	GLY825	−4.58
	Hydrophobic bond	ALA1042, TYR841, PRO840, LEU839, TRP926	
	Polar bond	THR1043, THR1044, ASN842	
	Charged bond	ARG1041, ARG838	
Alliin with 3zxr	Hydrogen bond	GLY307	−3.09
	Hydrophobic bond	LEU310, PRO314, LEU340, PRO417, PRO397	
	Polar bond	HID311, HID312, GLN398	
	Charged bond	ARG429	
Aloin with 3zxr	Hydrophobic bond	MET263, PRO266, LEU267, VAL142, PHE144, TYR153, VAL463, VAL324	−5.41
	Polar bond	SER143, HIS468, HIS468, ASN325	
	Charged bond	LYS265, LYS328, GLU154	
EMB with 3zxr	Hydrophobic bond	VAL155, TRP162, ALA170	−5.53
	Polar bond	ASN163	
	Charged bond	GLU154, ASP156	
ISN with 3zxr	Hydrogen bond	GLY177	−5.08
	Hydrophobic bond	TYR178, PRO174, PRO191	
	Polar bond	ASN175, GLN194	
	Charged bond	LSY179, ARG176, GLU169	
Octyl-β-d-glucopyranoside with 3zxr	Hydrophobic bond	VAL155, TYR153, PRO266, LEU267, PHE268, TRP162	−3.63
	Polar bond	HIS182	
	Charged bond	GLU154	
Oleanolic acid with 3zxr	Hydrophobic bond	PHE10, ALA13, VAL18, PHE42, ALA39	−7.97
	Charged bond	LYS14, LYS17	
Phytol with 3zxr	Hydrogen bond	GLY131	−4.74
	Hydrophobic bond	TRP282, TYR230, PHE232, ALA132, TYR129	
	Polar bond	HIS278, SER280, ASN229	
	Charged bond	GLU133, ARG347, ARG352, ARG364, GLU214, LYS215	
Alliin with 4ow8	Hydrogen bond	GLY191	−4.75
	Hydrophobic bond	ALA194	
	Polar bond	GLN188, HID192	
	Charged bond	ASP193, LYS164, ARG140	
Aloin with 4ow8	Hydrogen bond	GLY100	−5.4
	Hydrophobic bond	VAL98, LEU97, LEU148, ILE19, ALA20	
	Polar bond	ASN99	
	Charged bond	GLU96, GLU29	
EMB with 4ow8	Hydrophobic bond	ALA194, VAL166, ALA168	−4.94
	Polar bond	GLN188	
	Charged bond	ASP193, LYS164, ASP167	
ISN with 4ow8	Hydrogen bond	GLY161	−5.48
	Hydrophobic bond	MET95, LEU93, ALA62, ILE162, PHE160, ILE157, VAL76, ALA74	
	Polar bond	THR65, THR158	
	Charged bond	GLU61, ASP159	
Octyl-β-d-glucopyranoside with 4ow8	Hydrogen bond	GLY191	−4.36
	Hydrophobic bond	VAL166, ALA168, ALA169, PRO170, VAL171, ALA194, MET176, VAL177, MET178	
	Polar bond	HIS192, GLN188	
	Charged bond	LYS164, ASP167	
Oleanolic acid with 4ow8	Hydrophobic bond	PRO216, PHE217, ALA218, PRO238, LEU237, PRO236, PRO235, TYR208	−9.38
		SER212	
	Polar bond	LYS214, ARG215, LYS228	
Phytol with 4ow8	Hydrogen bond	GLY191, GLY161	−5.3
	Hydrophobic bond	ALA194, ILE184, VAL171, PRO170, ALA169, ALA168, ALA165, MET176, VAL177	
	Polar bond	GLN188, HIS192	
	Charged bond	ASP193, ASP167, LYS164, GLU61, ARG57, ARG140	
Alliin with 5kwa	Hydrogen bond	GLY159	−4.01
	Hydrophobic bond	VAL158, VAL222	
	Polar bond	THR224	
	Charged bond	GLU160, LYS225, ASP223	
Aloin with 5kwa	Hydrophobic bond	PRO191, LEU192, ILE193, PHE230, PRO234, LEU214	−4.0
	Charged bond	GLU231, ARG232, ARG212	
EMB with 5kwa	Hydrophobic bond	LEU241, LEU243	−4.58
	Polar bond	ASN331	
	Charged bond	ASP240, GLU244, GLU245, GLU336, LYS333	
ISN with 5kwa	Hydrophobic bond	PRO191, LEU192, PRO211	−3.7
	Charged bond	ARG232, GLU231, ARG212	
Octyl-β-d-glucopyranoside with 5kwa	Hydrogen bond	GLY296, GLY298, GLY516	−2.61
	Hydrophobic bond	CYS297, LEU301, ALA517	
	Polar bond	THR300, ASN416	
	Charged bond	LYS299, ASP371, GLU372	
Oleanolic acid with 5kwa	Hydrophobic bond	PRO285, LEU283, PRO271	−6.8
	Polar bond	SER282	
	Charged bond	ARG284, ARG279, GLU407, LYS275	
Phytol with 5kwa	Hydrophobic bond	LEU561, PRO562, PRO567, TRP570, ILE599	−3.02
	Polar bond	ASN563, THR565, ASN566, THR601	
	Charged bond	ASP560, ARG597, ASP600	

## Data Availability

Data is available within the article.

## Supplementary Materials

The following are available online at https://www.mdpi.com/article/10.3390/antiox10121990/s1, Table S1: The binding energy of interacted residues of receptors proteins of *M. tuberculosis* with diverse Phytomolecules.

## Abbreviations

MDR	multi-drug resistance
SMs	secondary metabolites
TB	tuberculosis
XDR	extensively drug resistance
TDR	total drug resistance
Mtb	*Mycobacterium tuberculosis*
DPPH	2,2-diphenyl-1-picryl-hydrazyl-hydrate
ABTS	2,2’-azino-bis(3-ethylbenzothiazoline-6-sulfonic acid
ISN	isoniazid
EMB	ethambutol
INH	isonicotinic acid hydrazide
ATCC	American Type Culture Collection
PDB	Protein Data Bank
MOA	mechanism of action
RMSD	root mean square deviation
