# Supplementary material for: Potential Anti-Mycobacterium tuberculosis Activity of Plant Secondary Metabolites: Insight with Molecular Docking Interactions
Source: Antioxidants (Basel). 2021 Dec 14;10(12):1990. doi: 10.3390/antiox10121990 (PMC8750514; doi:10.3390/antiox10121990)
Supplement: Supplementary file 1 [file antioxidants-10-01990-s001.zip › antioxidants-1430985-supplementary.pdf]

Table S1. The binding energy of interacted residues of receptors proteins of *M. tuberculosis* with diverse phytomolecules.

**Interactions with 3zxr**	**Binding Energy (kcal/mol)**
**Aloin**	−6.28
ISN	−5.424
Oleanolic acid	−5.048
Octyl-β-d- Glucopyranoside	−3.122
Alliin	−2.421
Phytol	−1.942
EMB	−0.938
**Interactions with 3pty**	**Binding Energy (kcal/mol)**
Aloin	−6.89
ISN	−6.375
Oleanolic acid	−4.27
Octyl-β-d- Glucopyranoside	−4.462
Alliin	−5.382
Phytol	−1.113
EMB	−2.963
**Interactions with 4ow8**	**Binding Energy (kcal/mol)**
Aloin	−5.209
ISN	−5.91
oleanolic acid	−3.628
Octyl-β-d- Glucopyranoside	−4.395
Alliin	−4.959
Phytol	−1.316
EMB	−2.66
